# Systemic lupus erythematosus induced by anti-tumour necrosis factor alpha therapy: a French national survey

**DOI:** 10.1186/ar1715

**Published:** 2005-03-01

**Authors:** Michel De Bandt, Jean Sibilia, Xavier Le Loët, Sebastian Prouzeau, Bruno Fautrel, Christian Marcelli, Eric Boucquillard, Jean Louis Siame, Xavier Mariette

**Affiliations:** 1 Rheumatology Department, Hôpital Robert Ballanger, Aulnay sous Bois, France; 2 CHU Hautepierre, Strasbourg, France; 3 Hôpital Bois-Guillaume, Rouen, France; 4 Hôpital de Saint Lo, Saint Lo, France; 5 Hôpital Pitié Salpétrière, Assistance Publique-Hôpitaux de Paris, Paris, France; 6 CHU côte de Nacre, Caen, France; 7 Hôpital de Saint Pierre de la Réunion, St Pierre France; 8 Polyclinique de Riaumont, Liévin, France; 9 Hôpital de Bicêtre, Assistance Publique-Hôpitaux de Paris, Le Kremlin Bicêtre, France

## Abstract

The development of drug-induced lupus remains a matter of concern in patients treated with anti-tumour necrosis factor (TNF) alpha. The incidence of such adverse effects is unknown. We undertook a retrospective national study to analyse such patients.

Between June and October 2003, 866 rheumatology and internal medicine practitioners from all French hospital centres prescribing anti-TNF in rheumatic diseases registered on the website of the 'Club Rhumatismes et Inflammation' were contacted by email to obtain the files of patients with TNF-induced systemic lupus erythematosus. Twenty-two cases were collected, revealing two aspects of these manifestations. Ten patients (six patients receiving infliximab, four patients receiving etanercept) only had anti-DNA antibodies and skin manifestations one could classify as 'limited skin lupus' or 'toxidermia' in a context of autoimmunity, whereas 12 patients (nine patients receiving infliximab, three patients receiving etanercept) had more complete drug-induced lupus with systemic manifestations and at least four American Congress of Rheumatology criteria. One patient had central nervous system manifestations. No patients had lupus nephritis. The signs of lupus occurred within a mean of 9 months (range 3–16 months) in patients treated with infliximab and within a mean of 4 months (range 2–5 months) in patients treated with etanercept. In all cases after diagnosis was determined, anti-TNF was stopped and specific treatment introduced in eight patients: two patients received intravenous methylprednisolone, four patients received oral steroids (15–35 mg/day), and two patients received topical steroids. Lupus manifestations abated within a few weeks (median 8 weeks, standard deviation 3–16) in all patients except one with longer-lasting evolution (6 months). At that time, cautious estimations (unpublished data from Schering Plough Inc. and Wyeth Inc.) indicated that about 7700 patients had been exposed to infliximab and 3000 to etanercept for inflammatory arthritides in France. It thus appears that no drug was more implicated than the other in lupus syndromes, whose incidence was 15/7700 = 0.19% with infliximab and 7/3800 = 0.18% with etanercept.

Clinicians should be aware that lupus syndromes with systemic manifestations may occur in patients under anti-TNF alpha treatment.

## Introduction

Therapy with anti-tumour necrosis factor (TNF) alpha is effective for rheumatoid arthritis (RA) [1,2], with an estimated 500,000 patients being treated worldwide. The possible occurrence of drug-induced autoimmune disorders remains a matter of concern [3]because induction of autoantibodies is frequently observed in patients treated with TNF alpha inhibitors [4]. Of concern is the possible induction of lupus-like (or drug-induced lupus) syndromes, but few cases have been reported [5-7]. In all reported cases, the signs disappeared after treatment was stopped. The incidence of cases is unknown.

We report here the results of a French national survey revealing 22 cases of drug-induced lupus erythematosus (systemic lupus erythematosus [SLE]) in French patients being treated with anti-TNF alpha for inflammatory arthritides.

## Methods

Between June and October 2003, the 'Club Rhumatismes et Inflammation', a section of the French Society of Rheumatology, carried out a retrospective survey among all French rheumatologists and specialists in internal medicine to uncover cases of SLE with anti-TNF alpha treatment (infliximab or etanercept at that time). Eight hundred and sixty-six rheumatology and internal medicine practitioners from all the French hospital centres prescribing anti-TNF in rheumatic diseases, registered on the website of the Club Rhumatismes et Inflammation , were contacted four times by email at 1-month intervals to obtain the files of patients with TNF-induced SLE. The study included all the patients ever known to have developed an SLE-like illness during anti-TNF treatment and not only those who developed an SLE-like illness during the 3-month study period.

As the prescription for anti-TNF alpha is limited to the hospitals in France, all the units of rheumatology using biologics were contacted. Eighteen units gave positive results, 22 gave negative results and very few (<10) did not participate. As all the units of rheumatology using biologics were contacted and most of them participated in the study, we can estimate that the survey involved almost all of the French patients treated with anti-TNF for arthritides. At that time, cautious estimations indicated that about 7700 patients had been exposed to infliximab and 3800 had been exposed to etanercept for inflammatory arthritides in France (unpublished data from Schering Plough Inc. and Wyeth Inc.).

As there are no recognized criteria for drug-induced lupus [8], we considered diagnosis in the case of: a patient with anti-TNF alpha treatment for inflammatory arthritides; a temporal relationship between clinical manifestations and anti-TNF alpha treatment; the presence of at least four American Congress of Rheumatology (ACR) criteria of SLE [9]. Musculoskeletal symptoms were taken into account only if they reappeared with other lupus symptoms in a patient in whom they had previously disappeared under anti-TNF therapy, and isolated positive results for antinuclear antibodies (ANA) or anti-dsDNA antibodies were not considered for diagnosis, given their high frequency in patients under this therapy. Telephone calls were made to collect information in the case of missing data. Physicians were asked to provide information about the clinical status of the patients and the presence of lupus criteria. Information about the immunological status of the patients was requested (before and after the onset of the manifestations as well as after drug discontinuation).

The biological tests used for the detection of autoantibodies were an indirect immunofluorescent assay for ANA, an ELISA or Farr assay for anti-DNA antibodies, Ouchterlony's method for anti-extractable nuclear antigens (anti-ENA), and an ELISA for anti-histone, anti-Ro, anti-La, anti-SM, anti-RNP, anti-JO1, anti-Topo 1, and anticardiolipin antibodies (ACL).

## Results

A total of 32 patients was collected, three of which had been previously described [5]. Ten patients were ruled out due to improper diagnosis of lupus syndrome, due to pre-existing lupus syndrome or due to mixed connective tissue disease before introduction of anti-TNF alpha therapies. We observed two types of manifestations among the remaining patients.

Ten patients (six patients treated with infliximab, four patients treated with etanercept) had a diagnosis of 'anti-TNF-induced SLE' based on three ACR criteria (Table 1). None of these patients had previous signs of lupus before treatment except one with isolated positive ANA. All of them had RA with joint erosions. The mean age at onset of RA was 39 years (range 24–57 years), and the mean disease duration before onset of 'SLE' was 13 years (range 6–31 years). All patients had been treated with a mean of five disease-modifying antirheumatic drugs, including methotrexate in all cases. Before anti-TNF therapy, no patients had clinical sign of lupus, one had positive isolated ANA (1/160) without any other lupus criteria, and no patients had anti-DNA or low complement. At the time of treatment, all patients were being treated with steroids (mean 8 mg/day, range 4–16 mg/day) and methotrexate. No patient had any other drug known as a lupus-inducing drug.

The only signs were isolated skin lesions (Table 2): pruritic rash (two cases), butterfly rash (three cases), photosensitivity (two cases), purpura (two cases), chilblains (one case), in a context of autoimmunity with positive ANA and anti-dsDNA antibodies. In all cases the clinical manifestations led to ceasing the treatment with anti-TNF alpha, and the signs abated quickly thereafter (<1 month). Despite the presence of three manifestations or criteria for systemic lupus we did not consider that these patients had drug-induced SLE, but rather that they presented toxidermia associated with ANA. Moreover, all of these clinical manifestations are not specific for lupus. Unfortunately no skin biopsy was performed.

Biological signs were positive results for ANA in all patients (new onset or rise in titre, range 1/160–1/250°; three patients with a speckled pattern, seven patients with a diffuse pattern). A new onset of positive results for anti-dsDNA antibodies in the 10 patients was noted by ELISA. None had any other biological and/or immunological manifestations of lupus. No confounding agent was involved in the occurrence of ANA and anti-dsDNA.

Twelve other patients (10 females, two males; nine patients receiving infliximab, three patients receiving etanercept) had a diagnosis of drug-induced systemic lupus supported by the presence of at least four ACR criteria (Tables 2 and 3). Eleven patients had erosive and destructive RA and one patient had severe psoriatic arthritis. The mean age at onset of RA was 36 years (range 14–54 years), and the mean disease duration before onset of SLE was 16 years (range 3–40 years). All patients had been treated with a mean of five disease-modifying antirheumatic drugs (range 2–8), including methotrexate in all cases.

Before anti-TNF therapy, no patients had clinical sign of lupus, three had positive ANA (range 1/160–1/1280), one of these (the patient with the highest level of ANA) had one time a limit positive anti-dsDNA titre (ELISA test, 46 UI; normal value <40), and nine had negative results. The two other patients with positive ANA had positive anti-Ro antibodies, and a clinical history of secondary Sjögren syndrome. None of the three patients with positive ANA had any other sign or lupus criteria (Table 2). Eleven patients had a typical history of severe and erosive RA and one patient a history of severe psoriatic arthritis. At the time of treatment, all patients were being treated with steroids (mean 9 mg/day; range 5–15 mg/day) and methotrexate (except one patient on etanercept alone).

Clinical signs were skin manifestations in 11 patients (papules, alopecia, rash, butterfly rash, photosensitivity), general manifestations in nine patients (fever, weight loss, asthenia), reappearance of polyarthritis in six patients, inflammatory myalgias in four patients, serositis in three patients, deep vein thrombosis (twice) in one patient, lung disease (life-threatening pneumonitis) with pleuritis in one patient, and neuritis of the third cranial nerve in one patient. No case of nephritis was found. The mean ACR criteria number was 5.5 (range 4–7).

Skin lesions were largely symmetrical (arm, face, trunk) and not at injection sites (in the case of etanercept). Histological analysis (four patients) revealed atrophy of the epidermis, necrosis of some keratinocytes, and perifollicular and perivascular lymphocytic infiltration in the dermis without vasculitis. No indirect immunofluorescent test was performed. The patient with deep vein thrombosis also had positive ACL antibodies. Articular symptoms were taken into account only if they reappeared with other lupus symptoms in a patient in whom they had previously disappeared under anti-TNF therapy and/or were different from previous complaints.

The patient with optical neuritis had no previous sign of multiple sclerosis before treatment with infliximab; she had an isolated neuritis of the third cranial nerve, with malar rash and autoantibodies. The lumbar puncture was normal. Magnetic resonance imaging showed an isolated hyper signal of the third cranial nerve. Extensive research for other manifestations of multiple sclerosis was performed without success. The presence of the neurological manifestations with other clinical signs and autoimmunity led to the diagnosis of drug-induced lupus.

Biological signs were positive results for ANA in all patients (new onset or rise in titre, range 1/160–1/2560°; four with a speckled pattern, eight with a diffuse pattern), and positive results for anti-dsDNA antibodies (new onset) in 11 patients by ELISA. Among the 11 patients tested with ELISA: five had anti-IgM antibodies and six had a positive test with no more detail; among them, three patients were tested by Farr assay and were positive. The patient without anti-DNA had a high titre of ANA, positive anti-ENA and anti-histone antibodies. Positive anti-ENA antibodies were present in five patients (two patients with previously known anti-SS-A/Ro antibodies, three patients with newly detected anti-ENA antibodies with an unidentified aspect), anti-histone in two patients and anti-cardiolipin in six patients. Leucopenia (blood count <4000/mm^3^), thrombopenia (blood count <100,000/mm^3^), lymphopenia (blood count <1500/mm^3^) and positive Coombs test (without haemolytic anaemia) were present in five patients, four patients, two patients and one patient, respectively. Elevated muscle enzymes were present in three of four patients with inflammatory myalgias. One patient had isolated elevated creatinin phosphokinase. No patient had muscle weakness. Transient low levels of C4 were detected in four cases (nine tested).

The signs of SLE occurred in a mean of 9 months in patients treated with infliximab and 4 months in patients treated with etanercept. In all cases, after the diagnosis was determined, the treatment was stopped and the manifestations then abated within a few weeks (median 8 weeks, range 3–16 weeks), except one (patient 12, Table 2) with a longer lasting evolution (6 months) before resolution. She had persistent asthenia, immunological and haematological abnormalities before resolution. But after 6 months all the signs abated. Biological signs normalized within a few months: in eight patients the ANA results were negative, and in four they were reduced; in nine patients, anti-dsDNA results were negative, and in three patients they were reduced.

Recovery was spontaneous without treatment in four cases. Steroids were needed in the eight other patients: two patients received topical steroids for skin lesions, two patients received intravenous methylprednisolone, and four patients received oral steroids (15–30 mg/day) for intense general signs. In no patient did SLE signs reappear.

Cautious estimations at that time (unpublished data from Schering Plough Inc. and Wyeth Inc.) indicated that about 7700 patients had been exposed to infliximab and 3800 patients had been exposed to etanercept for inflammatory arthritides in France. The incidence of lupus syndromes was thus the same with infliximab (15/7700 = 0.19%) and with etanercept (7/3800 = 0.18%).

## Discussion

We report on 22 patients treated with anti-TNF alpha for severe RA or psoriatic arthritis (15 patients receiving infliximab and seven patients receiving etanercept) with no previous sign of lupus disease who developed clinical and biological manifestations of drug-induced lupus.

We are aware that the scientific interest of a retrospective analysis is of limited value compared with a prospective study. However, at that time only isolated case reports were available. As far as we know, this survey is the only one providing further information about the clinical problem of drug-induced lupus. We hope that the national observatories and registers that have been settled in various countries around the world will precisely and prospectively answer the question of anti-TNF-induced lupus.

Analysis of the cases revealed two subgroups of patients. The first group of patients was considered by the referring physician as 'drug-induced lupus'. In our opinion these patients had what we term 'toxidermia' – that is, isolated skin manifestations in a context of autoimmunity and the absence of systemic manifestations. We are aware that some colleagues will feel uneasy with the term 'toxidermia' and would prefer to qualify these patients as 'incomplete lupus with isolated skin manifestations'. We do understand the reserve about the expression 'toxidermia' rather than 'drug-induced lupus erythematosus'. We preferred to be rigorous with the diagnosis of SLE and to use a more stringent definition (at least four ACR criteria for SLE) to describe a core of patients. Indeed, patients treated with anti-TNF (mostly infliximab rather than etanercept) have frequent and isolated skin manifestations with positive autoantibodies. The frequency of these clinical pictures is unknown but seems important regarding the frequency of autoantibodies (up to 50% for ANA, 25% for ACL and 15% for anti-DNA with infliximab) and of skin manifestations [3-7,10-12]. Do all these patients only have toxidermia in a context of autoimmunity or 'limited drug-induced skin lupus'?

The second group of 12 patients had what we considered true 'drug-induced SLE' with at least four ACR criteria and systemic manifestations, with a very acute and complete syndrome, associating general manifestations and clinical and biological signs of lupus. All 12 patients fulfilled the ACR criteria for SLE [9], and did not simply have drug-induced skin toxidermia in a context of autoimmunity. Clinicians should thus be aware that lupus syndromes may occur in patients under anti-TNF alpha treatment and may be complicated by central nervous system signs. However, withdrawal of the drug leads to an abatement of the signs.

ACL antibodies were detected in six patients whereas only one patient had developed thrombosis. The occurrence of ACL antibodies in anti-TNF alpha-treated patient is well documented [13]: up to 25% of RA patients with anti-TNF develop IgG or IgM ACL, but thrombosis is observed in much fewer patients (about 4%). It is also known that TNF has potent anti-thrombotic properties [14]. It is therefore conceivable that the association of ACL antibodies and inhibition of TNF could lead to an increase number of thrombosis. Should we routinely look for ACL antibodies in our patients?

The imputability of anti-TNF therapy in inducing lupus syndrome is probable, given the temporal relationship between the onset of signs with treatment and the resolution following withdrawal of the drug in all cases. No confounding agent (such as statins) was involved in patients with myositis, myalgias or elevated creatinin phosphokinase. No other drug, known as a lupus-inducing-drug, was present in any patient.

The incidence of anti-TNF-induced lupus is difficult to evaluate. We estimated that we covered most, if not all, cases in France as of October 2003. It is possible we have missed some cases, as in all retrospective studies. However, with the opportunity to use the quite unique organized system that is the Club Rhumatismes et Inflammation website, which encompasses most if not all the physicians interested in biologics and systemic diseases, we think these missing cases are scarce. Moreover, we sent four email recall letters at 1-week intervals to detect the cases. The estimation of the number of exposed patients to the drugs is always difficult, even by the pharmaceutical companies themselves. At the time, cautious estimations from Schering Plough Inc. and Wyeth Inc. allowed one to determine the number of exposed patients to each of the drugs from the beginning of the clinical trials until the time of the study, but did not allow one to determine the length of exposition in terms of the number of patients-years. Therefore, with these estimations, it appears that no drug was more implicated than the other in lupus syndromes.

Interestingly, no case of lupus nephritis was observed in this survey. However, one case of etanercept-associated renal disease has been recently described (active urine sediment, new onset of anti-Ro, anti-Sm and anti-RNP antibodies) but no biopsy has been performed. In that case signs abated shortly after drug discontinuation [15].

The mechanism of induction remains unclear. One hypothesis could be an increase of apoptotic particles and antigens from apoptotic cells. It has been shown that RA patients had no circulating nucleosomes at the steady state and some of them had significantly higher levels of plasma nucleosomes after receiving infliximab [16]. The accumulation of nucleosomes could possibly enhance the development of autoantibodies in subjects with appropriate genetic backgrounds.

Another hypothesis is that the suppression of the T-helper type 1 response by TNF blockers could favour a T-helper type 2 response leading to SLE, but this hypothesis needs to be tested in man. Neutralization of TNF alpha was tested in mice undergoing acute graft versus host disease using the parent-into-F1 model [17]. Monoclonal antibody against TNF alpha blocked the lymphocytopenic features characteristic of acute graft versus host disease and induced a lupus-like chronic graft versus host disease phenotype (lymphoproliferation and autoantibody production). These effects resulted from complete inhibition of detectable anti-host cytotoxic T lymphocytes. In that model the authors showed that *in vivo *blockade of TNF alpha preferentially inhibited the production of interferon gamma and blocked interferon-gamma-dependent upregulation of Fas; and that cytokines such as IL-10, IL-6, or IL-4 were not inhibited. These results suggest that a therapeutic TNF alpha blockade may promote humoral autoimmunity by selectively inhibiting the induction of a cytotoxic T lymphocyte response that would normally suppress autoreactive B cells.

A final hypothesis is the role of bacterial infections. They are increased with TNF blockers and are also powerful stimulants leading to polyclonal B-lymphocyte activation and autoantibody production. Some cases of positive anti-DNA following infection after etanercept have been reported [18]. Interestingly, the titre returned to normal values after antibiotic treatment.

In conclusion, we collected 22 cases of 'anti-TNF alpha-induced lupus' based on the ACR lupus criteria in a retrospective national survey, allowing us to better define the clinical aspects of these manifestations. Given the frequency of autoantibodies in anti-TNF alpha-treated patients, we proposed to identify two subsets of patients. The first group only had skin manifestations and anti-DNA antibodies. Do these patients have 'toxidermia' in a context of autoimmunity or true 'limited drug-induced skin lupus'? Should they stop or continue treatment with anti-TNF alpha? We do not have the answer, and we let the reader decide. Whereas the second group has true drug-induced SLE (with at least four ACR criteria) and systemic manifestations (serositis, cranial neuritis). In all cases anti-TNF was stopped after diagnosis was determined, and specific manifestations abated within a few weeks. Clinicians should be aware that lupus syndromes with systemic manifestations may occur in patients receiving anti-TNF alpha treatment.

## Abbreviations

ACL = anticardiolipin antibodies; ACR = American Congress of Rheumatology; ANA = antinuclear antibodies; ELISA = enzyme-linked immunosorbent assay; ENA = extractable nuclear antigens; IL = interleukin; RA = rheumatoid arthritis; SLE = systemic lupus erythematosus; TNF = tumour necrosis factor.

## Competing interests

The author(s) declare that there are no competing interests.

## Authors' contributions

MDB had the original idea for the work, collected all the data, analysed the data and wrote the paper. JS, XLL, SP, BF, ChM, EB and JLS were all in charge of the patients, reviewed the charts, and gathered and collected the data. XM participated in the data analysis and the writing of the work.

## Figures and Tables

**Table 1:** General presentation of the 10 patients with 'limited skin lupus' or toxiderma in a context of autoimmunity

Patient	Before anti-TNF alpha treatment				During onset of symptoms		
	
	Disease	Autoantibody	Clinical signs of lupus	Treatment	Duration of treatment (months)	Clinical signs of lupus	Biological signs of lupus
1	RA, erosive	RF+	None	ETA	36	Skin	ANA+, dsDNA+
2	RA, erosive	RF+	None	INF	6	Skin	ANA+, dsDNA+
3	RA, erosive	RF+, ANA+ 1:160°	None	INF	18	Skin	ANA+, dsDNA+
4	RA, erosive	RF-	None	ETA	5	Skin	ANA+, dsDNA+
5	RA, erosive	RF+	None	INF	7	Skin	ANA+, dsDNA+
6	RA erosive	RF+	None	ETA	5	Skin	ANA+, dsDNA+
7	RA, erosive	RF+	None	INF	11	Skin	ANA+, dsDNA+
8	RA, erosive	RF+	None	INF	3	Skin	ANA+, dsDNA+
9	RA, erosive	RF+	None	ETA	12	Skin	ANA+, dsDNA+
10	RA, erosive	RF+	None	INF	13	Skin	ANA+, dsDNA+

ANA, antinuclear antibodies; dsDNA, double-strand DNA; ETA, etanercept; INF, infliximab; RA, rheumatoid arthritis; RF, rheumatoid factor; TNF, tumour necrosis factor; +, positive; -, negative.

**Table 2:** General presentation of the 12 patients with 'complete lupus'

Patient	Before anti-TNF alpha treatment				During onset of symptoms		
	
	Disease	Autoantibody	Clinical signs of lupus	Treatment	Duration of treatment (months)	Clinical signs of lupus^a^	Biological signs of lupus
1	RA, RF+, erosive	None	None	INF	27	General, skin, serositis, lung	ANA+, dsDNA+, ACL+, leucopenia, thrombopenia, ENA+
2	RA, RF+, erosive	ANA+, Ro+	None	INF	4	General, skin (3), arthritis	ANA+, dsDNA+, histone +
3	RA, RF+, erosive	ANA+, Ro+	None	INF	2	Skin, myalgias, arthritis	ANA+, dsDNA+, ENA+,
4	RA, RF-, erosive	None	None	ETA	4	General, skin (2), myositis	ANA+, dsDNA+, ACL+, low C4, ENA+
5	RA, RF+, erosive	None	None	INF	4	Skin (3), myositis, arthritis, pericarditis	ANA+, dsDNA+, ACL+, CPK, lymphopenia
6	RA, RF+, erosive	ANA+, dsDNA+ limit value	None	ETA	5	General, skin	ANA+, dsDNA+, thrombopenia, leucopenia
7	RA, RF+, erosive	None	None	INF	10	General, serositis, myositis	ANA+, dsDNA+, ACL+, leucopenia, thrombopenia, CPK,
8	RA, RF+	None	None	ETA	2	Phlebitis, skin	ANA+, dsDNA+, leucopenia, ACL+, thrombopenia
9	Psoriatic arthritis	None	None	INF	14	General, skin, neurological	ANA+, dsDNA+
10	RA, RF+, erosive	None	None	INF	16	General, Skin, arthritis	ANA+, dsDNA+
11	RA, RF+, erosive	None	None	INF	12	General, skin (3), arthritis, myositis	ANA+, dsDNA+, ENA+, CPK
12	RA, RF+, erosive	None	None	INF	10	General, skin, arthritis	ANA+, dsDNA+, CPK, ENA+, ACL+, low C4, histone+, leucopenia, lymphopenia, Coombs test+

ACL, positive anticardiolipin antibodies; ANA, antinuclear antibodies; CPK, creatin phospokinase or muscle enyme, elevated muscle enzymes; dsDNA, double-strand DNA; ENA, positive anti-extractable nuclear antigens antibodies; ETA, etanercept; general, general manifestations (fever, weight loss, asthenia); histone, positive anti-histone antibodies; INF, infliximab; RA, rheumatoid arthritis; RF, rheumatoid factor; TNF, tumour necrosis factor; +, positive; -, negative.

^a^The skin manifestations were as follows (number in parentheses indicates number of skin manifestations observed in one patient): maculo papular rash observed in six patients, butterfly rash observed in five patients, alopecia present in one patient, photosensitivity observed in five patients, purpuric lesions observed in two patients. One patient had no skin manifestations, seven patients had one sign, one patient had two different signs, and three patients had three skin manifestations.

**Table 3:** Signs of systemic lupus erythematosus (SLE) in 12 patients under anti-tumour necrosis factor alpha treatment

Sign of SLE	Number of patients
Skin (≥ 1 SLE skin criteria)	11
General (fever, weight loss, asthenia)	9
Musculoskeletal (arthritis, myositis, myalgias)	10 (6 arthritis, 3 myositis, 1 myalgia)
Serositis, lung, neuritis, phlebitis; haematological abnormalities, elevated muscle enzymes	3, 1, 1 and 1, respectively; 12 and 3, respectively
Antibody testing	
Positive for ANA (>1/160)	12
Positive for anti-dsDNA antibodies (>40 UI)	11
Positive anti-ENA, anti-histone, anti-cardiolipid	5, 2 and 6, respectively
Recovery	
After treatment withdrawn (with/without steroids)	12 (4 and 8)
Delay in recovery (median and range)	8 weeks (3–16 weeks)

Ten females, two males; nine patients treated with infliximab and three patients treated with etanercept. ANA, antinuclear antibodies; dsDNA, double-strand DNA; ENA, positive anti-extractable nuclear antigensantibodies.

## References

[B1] Garrison L, McDonnell N (1999). Etanercept: therapeutic use in patients with rheumatoid arthritis. Ann Rheum Dis.

[B2] Maini R, St Clair EW, Breedveld F, Furst D, Kalden J, Weisman M, Smolen J, Emery P, Harriman G, Feldmann M, Lipsky P (1999). Infliximab (chimeric anti-tumour necrosis factor alpha monoclonal antibody) versus placebo in rheumatoid arthritis patients receiving concomitant methotrexate: a randomised phase III trial. ATTRACT Study Group. Lancet.

[B3] Pisetski DS (2000). Tumor necrosis factor alpha blockers and the induction of anti-DNA antibodies. Arthritis Rheum.

[B4] Louis M, Rauch J, Armstrong M, Fitzcharles M-A (2003). Induction of autoantibodies during prolonged treatment with infliximab. J Rheumatol.

[B5] De Bandt M, Vittecoq O, Descamps V, Le Loet X, Meyer O (2003). Anti-TNF alpha-induced systemic lupus erythematosus. Clin Rheumatol.

[B6] Shakoor N, Michalska M, Harris CA, Block JA (2002). Drug-induced systemic lupus erythematosus associated with etanercept therapy. Lancet.

[B7] De Rycke L, Kruithof E, Van Damme N, Hoffman IEA, Van den Bossche N, Van den Bosch F, Veys EM, De Keyser F (2003). Antinuclear antibodies following infliximab treatment in patients with rheumatoid arthritis or spondylarthropathy. Arthritis Rheum.

[B8] Mongey AB, Hess E, Lahita R (1999). Drug and environmental lupus: clinical manifestations and differences. Systemic Lupus Erythematosus.

[B9] Hochberg MC (1997). Updating the American College of Rheumatology revised criteria for the classification of systemic lupus erythematosus. Arthritis Rheum.

[B10] Schaible TF (2001). Treatment of inflammatory diseases: safety of long-term use of infliximab. Presse Med.

[B11] Devos SA, Van Den Bossche N, De Vos M, Naeyaert JM (2003). Adverse skin reactions to anti-TNF-alpha monoclonal antibody therapy. Dermatology.

[B12] Flendrie M, Creemers MC, Welsing PM, den Broeder AA, van Riel PL (2003). Survival during treatment with tumour necrosis factor blocking agents in rheumatoid arthritis. Ann Rheum Dis.

[B13] Jonsdottir T, Forslid J, van Vollenhoven A, Harju A, Brannemark S, Klareskog L, van Vollenhoven RF (2004). Treatment with tumour necrosis factor alpha antagonists in patients with rheumatoid arthritis induces anticardiolipin antibodies. Ann Rheum Dis.

[B14] Cambien B, Bergmeier W, Saffaripour S, Mitchell HA, Wagner DD (2003). Antithrombotic activity of TNF-alpha. J Clin Invest.

[B15] Carlson E, Rothfield N (2003). Etanercept-induced like lupus syndrome in a patient with rheumatoid arthritis. Arthritis Rheum.

[B16] D'Auria F, Rovere-Querini P, Giazzon M, Ajello P, Baldissera E, Manfredi A, Sabbadini M (2004). Accumulation of plasma nucleosomes upon treatment with anti-tumour necrosis factor-alpha antibodies. J Intern Med.

[B17] Via CS, Shustov A, Rus V, Lang T, Nguyen P, Finkelman FD (2001). In vivo neutralization of TNF-alpha promotes humoral autoimmunity by preventing the induction of CTL. J Immunol.

[B18] Ferraccioli G, Mecchia F, Di Poi E, Fabris M (2002). Anticardiolipin antibodies in rheumatoid patients treated with etanercept or conventional combination therapy: direct and indirect evidence for a possible association with infections. Ann Rheum Dis.

